# Evaluation of the association of birth order and group childcare attendance with Kawasaki disease using data from a nationwide longitudinal survey

**DOI:** 10.3389/fped.2023.1127053

**Published:** 2023-03-28

**Authors:** Takahiro Namba, Akihito Takeuchi, Naomi Matsumoto, Mitsuru Tsuge, Masato Yashiro, Hirokazu Tsukahara, Takashi Yorifuji

**Affiliations:** ^1^ Department of Pediatrics, Fukuyama City Hospital, Fukuyama, Japan; ^2^ Department of Pediatrics, Okayama University Graduate School of Medicine, Dentistry and Pharmaceutical Sciences, Okayama, Japan; ^3^ Department of Neonatology, National Hospital Organization Okayama Medical Center, Okayama, Japan; ^4^ Department of Epidemiology, Okayama University Graduate School of Medicine, Dentistry and Pharmaceutical Sciences, Okayama, Japan

**Keywords:** Kawasaki disease (KD), birth order, group childcare, infectious diseases, vasculitis

## Abstract

**Background:**

Kawasaki disease (KD) is a form of pediatric systemic vasculitis. Although the etiology remains unclear, infections have been identified as possible triggers. Children with a later birth order and those who attend childcare are at a higher risk of infections due to exposure to pathogens from their older siblings and other childcare attendees. However, longitudinal studies exploring these associations are limited. Thus, we aimed to elucidate the relationship between birth order, group childcare attendance, and KD, using a nationwide longitudinal survey in Japan.

**Methods:**

In total, 36,885 children born in Japan in 2010 were included. The survey used questionnaires to identify hospitalized cases of KD. We evaluated the relationship between birth order classification, group childcare attendance, and KD prevalence every year, from 6 to 66 months of age. For each outcome, odds ratios (ORs), and 95% confidence intervals (CIs) were estimated after adjusting for child factors, parental factors, and region of residence.

**Results:**

Children with higher birth orders were more likely to be hospitalized with KD at 6–18 months of age (second child OR: 1.77, 95% CI: 1.25–2.51; third child OR: 1.70, 95% CI: 1.08–2.65). This trend was stronger for children who did not attend group childcare (second child OR: 2.51, 95% CI: 1.57–4.01; third child OR: 2.41, 95% CI: 1.30–4.43). An increased risk of KD hospitalization owing to the birth order was not observed in any age group for children in the childcare group.

**Conclusions:**

Children with higher birth orders were at high risk for hospitalization due to KD at 6–18 months of age. The effect of birth order was more prominent among the children who did not attend group childcare.

## 1. Introduction

Kawasaki disease (KD) is an acute systemic vasculitis that is primarily observed in the pediatric population (1–5). KD can be attributed to both infectious and genetic factors (3–5), in that infections can trigger an overactive immune system in genetically susceptible individuals (2–4). KD symptoms are similar to those of some infectious diseases and include fever, conjunctival hyperemia, rash, edema of the extremities, and lymphadenitis (6, 7). Staphylococcus aureus and Streptococcus pyogenes have been reported as possible bacterial triggers of KD (8), whereas adenovirus, herpesvirus, and parainfluenza virus are viral triggers of KD (8). SARS-CoV-2, the cause of the recent pandemic, has also been reported to cause KD or multisystem inflammatory syndrome in children, which is a disease that overlaps with KD (9).

In Japan, epidemiological surveys of patients with KD have been conducted every two years since 1970. Three significant epidemics were recorded in 1979, 1982, and 1986 (10). Furthermore, the geographical migration of epidemics and outbreaks in small areas has been observed (2, 10). KD appears to have seasonal trends (2, 7, 8, 10), with peaks noted from spring to summer in China and from winter to spring in Japan, the United Kingdom, Australia, and the United States (2, 7, 8, 10). Based on these epidemiological observations, it has been suggested that KD may be associated with infectious triggers.

Younger children are considered to be more susceptible to the pathogens carried by their older siblings and are thus more likely to develop infections (11, 12). In addition, children in childcare programs may be exposed to pathogens at childcare centers (11, 13, 14). Therefore, the significant impact of birth order and group childcare on the natal environment has been the subject of many studies. However, there are only a few reports on the relationship between these factors and KD (15). Therefore, in this study, we aimed to examine the association between birth order, group childcare, and KD development using data from a nationwide Japanese birth cohort.

## 2. Materials and methods

### 2.1. Study participants

**Figure 1:**
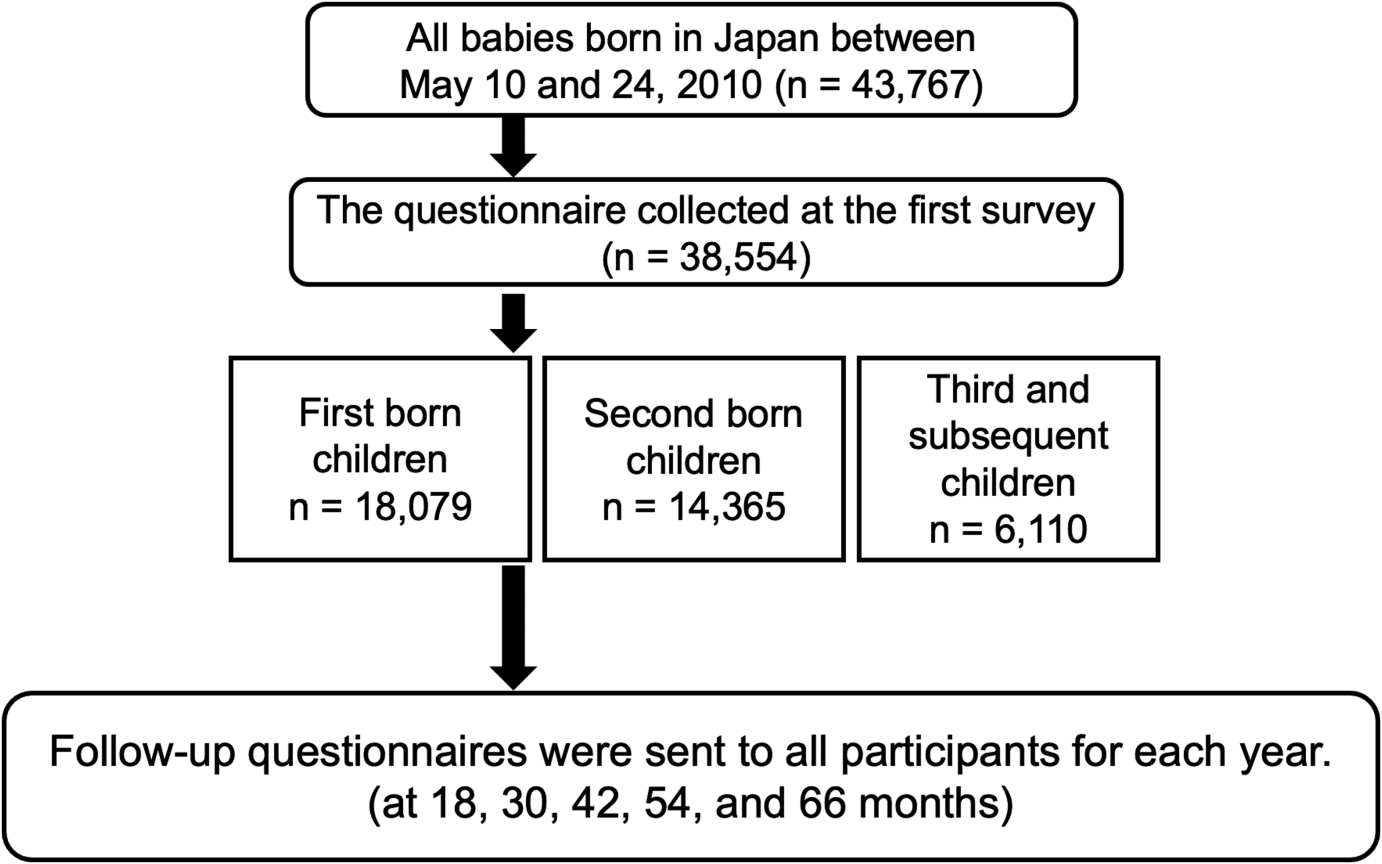
Flowchart showing data collection from the study participants. A baseline questionnaire was administered to all households when infants born during the study period were 6 months old. Subsequently, follow-up questionnaires were sent to all initial survey respondents at 1-year intervals (18, 30, 42, 54, and 66 months).

We used data from the Longitudinal Survey of Newborns in the 21st Century (2010 cohort), which was conducted on newborns born in Japan between May 10 and May 24, 2010, under the auspices of the Japanese Ministry of Health, Labour and Welfare (MHLW). A baseline questionnaire (Supplementary File S1) was administered to all households when infants born during the study period were 6 months old. Of the total 43,767 questionnaires mailed, 38,554 questionnaires were successfully collected (88.1% response rate). Subsequently, follow-up questionnaires (Supplementary File S2) were sent to all initial survey respondents at 1-year intervals (18, 30, 42, 54, and 66 months) (Figure 1), with the final survey being completed in 2015. In addition, birth record data (sex, singleton/multiple births, gestational age, and maternal parity) from the Japanese Vital Statistics database were linked for each surveyed child. The Vital Statistics are basic statistics conducted by the MHLW and include information on births, deaths, stillbirths, marriages, and divorces in Japan during the survey year.

### 2.2. Outcome measures

The survey asked whether the child had been hospitalized for any reason in the previous 12 months. The specific questions were set as follows: “During the past year, have you had any illnesses or injuries for which you have been seen in a hospital or clinic? Please circle all numbers that apply. Of the ailments you circled, please indicate below the number of each illness or injury that required hospitalization.” The same question was asked in each survey at age intervals of 6–18, 18–30, 30–42, 42–54, and 54–66 months, and parents selected the cause of the hospitalization from a list of specific disease name options. If the reason for hospitalization was KD, the child was considered to have contracted KD during the relevant period.

### 2.3. Exposure variables

We used the number of living children of the mothers at birth as an indicator of birth order. Parity information obtained from 38,554 participants was included in the analysis. The survey asked respondents about their usual caregivers of their children. Children for whom the response was from a group childcare provider were assumed to attend group childcare.

### 2.4. Covariates

Covariates were selected with reference to previous studies (16–18) and included child and parental factors, and the region of residence. Child factors included sex, singleton/multiple births, full-term/preterm birth (<37 weeks of gestation), and maternal parity. Parental factors included the maternal age at delivery, the mother's smoking habits (non-smoker, smoker of <10 cigarettes per day, and smoking of >10 cigarettes per day), and both parents' educational attainment (junior high school and others, high school, junior college [2 years] or vocational school, and university [4 years] or higher). The mother's smoking status was established through the initial survey (at six months), while the parents' educational attainments, which was an indicator of socioeconomic status, were taken from the second survey. The region of residence referred to the participants' place of origin (ward, city, town, or village) and was taken from the 2010 census. In the main analysis, we performed a multivariate analysis with the presence or absence of group childcare at the age of 18 months as an adjustment variable.

### 2.5. Statistical analysis

We evaluated the relationship between birth order classifications (first, second, third, or higher) and KD prevalence at each time point between six and 66 months of age. We analyzed the relationship between birth order and hospitalization for KD using a logistic regression model. In a subgroup analysis, we examined whether the relationship changed with group childcare during early infancy. Patients with missing data were excluded. Odds ratios (ORs) and 95% confidence intervals (95% CIs) were estimated for the outcomes. The oldest child category was used as the reference group throughout the analysis. We adjusted for potential confounders, such as child and parental factors and region of residence. Stata SE version 17 statistical software (Stata Corp., College Station, TX, United States) was used for analysis.

### 2.6. Ethics statement

Written consent was obtained from the parents of all the study participants. This study was approved by the Institutional Review Board of Okayama University Graduate School of Medicine, Dentistry, and Pharmaceutical Sciences (No. 1506-073).

## 3. Results

The baseline characteristics of the children in the study, categorized by birth order, are summarized in Table 1. The first child was the most common birth order, followed by the second and subsequent children (Table 1). As birth order increased, multiple births, premature births, and the use of childcare centers increased. The third and subsequent children were more likely to have mothers who were smokers, parents with lower education levels, and births in rural areas (towns and villages) compared to first and second children.

**Table 1:** Demographic characteristics of children in the study by birth order.

	First *N* = 18,079		Second *N* = 14,365		Third *N* = 6,110	
Sex, *n* (%)						
Male	9,324	(51.6%)	7,322	(51.0%)	3,198	(52.3%)
Female	8,755	(48.4%)	7,043	(49.0%)	2,912	(47.7%)
Singleton birth, *n* (%)	17,891	(99.0%)	14,048	(97.8%)	5,892	(96.4%)
Multiple births, *n* (%)	188	(1.0%)	317	(2.2%)	218	(3.6%)
Term birth, *n* (%)	17,174	(95.0%)	13,605	(94.7%)	5,677	(92.9%)
Preterm birth, *n* (%)	905	(5.0%)	760	(5.3%)	433	(7.1%)
Group childcare attendance at 1.5 years old						
Not attending, *n* (%)	11,508	(73.5%)	9,037	(72.1%)	3,557	(69.0%)
Attending, *n* (%)	4,144	(26.5%)	3,505	(27.9%)	1,596	(31.0%)
Maternal age at delivery (years), mean (SD)	30.2	(5.0)	32.0	(4.5)	33.8	(4.3)
Maternal smoking status, *n* (%)						
Non-smoker	17,042	(94.5%)	13,347	(93.2%)	5,368	(88.2%)
Smoker (≤10 cigarettes per day)	785	(4.4%)	739	(5.2%)	512	(8.4%)
Smoker (>10 cigarettes per day)	203	(1.1%)	239	(1.6%)	209	(3.4%)
Maternal educational attainment, *n* (%)						
University or higher	4,815	(30.8%)	3,137	(25.1%)	836	(16.3%)
Junior college or vocational school	6,262	(40.1%)	5,366	(42.8%)	2,059	(40.1%)
High school	3,871	(24.8%)	3,447	(27.5%)	1,800	(35.0%)
Junior high school and others	667	(4.3%)	574	(4.6%)	443	(8.6%)
Paternal educational attainment, *n* (%)						
University or higher	7,302	(47.8%)	5,385	(43.5%)	1,703	(33.7%)
Junior college or vocational school	2,805	(18.4%)	2,275	(18.4%)	917	(18.2%)
High school	4,238	(27.8%)	3,915	(31.6%)	1,900	(37.7%)
Junior high school and others	920	(6.0%)	817	(6.5%)	525	(10.4%)
Residential area, *n* (%)						
Wards	5,677	(31.4%)	3,913	(27.2%)	1,418	(23.2%)
Cities	11,113	(61.5%)	9,254	(64.4%)	4,039	(66.1%)
Towns or villages	1,289	(7.1%)	1,198	(8.4%)	653	(10.7%)

The data are expressed as numbers, *n* (%). For maternal age at delivery, age (median) and standard deviation (SD) are expressed.

The follow-up rates of the surveyed children for each period are presented in Table 2. The follow-up rate for the sixth survey was 68.8%. The percentage of hospitalizations due to KD as well as ORs and 95% CIs for the association between hospitalization due to KD and birth order between 6 and 66 months of age are also shown in Table 2. After adjusting for potential confounding variables, the likelihood of hospitalization due to KD was higher for the second and subsequent children than for the first child between 6 and 18 months of age (second child OR: 1.77, 95% CI: 1.25–2.51; third child OR: 1.70, 95% CI: 1.08–2.65). Birth order was not associated with hospitalization for KD at 18–30 months, 30–42 months, 42–54 months, and 54–66 months. In this analysis, we performed a multivariate analysis with the presence or absence of group childcare at the age of 18 months as an adjustment variable. There was no significant increase in the odds ratio with or without group childcare in any age group.

**Table 2:** Association between birth order and hospitalization due to Kawasaki disease among Japanese children between 6 and 66 months of age.

Age interval [*n*; response rate (%)]; Birth order	*N* case/*N* (%)	Odds ratio (95% CI)
Between 6 and 18 months (*n* = 32,035; 83.1%)		
First	54/15,000 (0.36%)	1 (ref.)
Second	77/12,108 (0.64%)	1.77 (1.25–2.51)
Third	30/4,927 (0.61%)	1.70 (1.08–2.65)
Between 18 and 30 months (*n* = 30,946; 80.3%)		
First	55/14,454 (0.38%)	1 (ref.)
Second	44/11,733 (0.38%)	0.99 (0.66–1.45)
Third	13/4,759 (0.27%)	0.72 (0.39–1.31)
Between 30 and 42 months (*n* = 28,173; 73.1%)		
First	40/13,144 (0.30%)	1 (ref)
Second	34/10,765 (0.32%)	1.04 (0.66–1.64)
Third	13/4,264 (0.30%)	1 (0.53–1.87)
Between 42 and 54 months (*n* = 26,591; 69.0%)		
First	25/12,521 (0.20%)	1 (ref)
Second	17/10,137 (0.17%)	0.84 (0.45–1.56)
Third	2/3,933 (0.05%)	0.25 (0.06–1.07)
Between 54 and 66 months (*n* = 26,529; 68.8%)		
First	29/12,538 (0.23%)	1 (ref)
Second	9/10,068 (0.09%)	0.39 (0.18–0.82)
Third	5/3,923 (0.13%)	0.55 (0.21–1.42)

Adjusted for child factors (sex, singleton/multiple births, gestational age, parity), parental factors (maternal smoking, maternal education, paternal education), and region of residence. The data are expressed as numbers, *n* (%). CI, confidence interval.

The subgroup analysis of the surveyed children stratified by group childcare status is presented in Table 3. After controlling for potential confounding variables, the likelihood of hospitalization due to KD between 6 and 18 months was higher for the second and subsequent children than for the first child (second child OR: 2.51, 95% CI: 1.57–4.01; third child OR: 2.41, 95% CI: 1.30–4.43). This trend was stronger when children in group childcare were excluded from the analysis. No association was observed between birth order and hospitalization for KD among children not in group care at 18–30 months, 30–42 months, 42–54 months, and 54–66 months. In addition, an increased risk of KD hospitalization due to birth order was not observed in any group for children in group childcare.

**Table 3:** Association between birth order and hospitalization due to Kawasaki disease among Japanese children with and without childcare attendance between 6 and 66 months of age.

Age interval [*n*; response rate (%)]; Birth order	*N* case/*N* (%)	Odds ratio (95% CI)
**No childcare attendance**		
Between 6 and 18 months (*n* = 22,972; 59.6%)		
First	29/10,923 (0.27%)	1 (ref.)
Second	56/8,670 (0.65%)	2.51 (1.57–4.01)
Third	21/3,379 (0.62%)	2.41 (1.31–4.43)
Between 18 and 30 months (*n* = 19,625; 50.9%)		
First	22/9,476 (0.23%)	1 (ref.)
Second	30/7,328 (0.41%)	1.57 (0.87–2.83)
Third	5/2,821 (0.18%)	0.75 (0.27–2.06)
Between 30 and 42 months (*n* = 15,546; 40.3%)		
First	20/7,587 (0.26%)	1 (ref)
Second	23/5,812 (0.40%)	1.49 (0.79–2.82)
Third	10/2,147 (0.47%)	2.09 (0.92–4.73)
Between 42 and 54 months (*n* = 9,816; 25.4%)		
First	6/4,751 (0.20%)	1 (ref)
Second	6/3,724 (0.17%)	1.29 (0.40–4.13)
Third	0/1,335 (0.05%)	1 (empty)
Between 54 and 66 months (*n* = 9,324; 24.2%)		
First	10/4,536 (0.23%)	1 (ref)
Second	2/3,521 (0.09%)	0.14 (0.18–1.13)
Third	1/1,267 (0.13%)	0.51 (0.06–4.25)
**With childcare attendance**		
Between 6 and 18 months (*n* = 9,054; 23.5%)		
First	25/4,074 (0.61%)	1 (ref.)
Second	21/3,433 (0.61%)	1.00 (0.54–1.83)
Third	9/1,547 (0.58%)	0.86 (0.37–1.98)
Between 18 and 30 months (*n* = 11,308; 29.3%)		
First	33/4,970 (0.23%)	1 (ref.)
Second	13/4,403 (0.41%)	0.50 (0.25–1.00)
Third	8/1,935 (0.18%)	0.68 (0.29–1.59)
Between 30 and 42 months (*n* = 12,561; 32.6%)		
First	20/5,527 (0.26%)	1 (ref)
Second	11/4,929 (0.40%)	0.55 (0.25–1.19)
Third	3/2,105 (0.47%)	0.35 (0.10–1.21)
Between 42 and 54 months (*n* = 16,748; 43.4%)		
First	19/7,752 (0.25%)	1 (ref)
Second	11/6,401 (0.17%)	0.78 (0.36–1.68)
Third	2/2,595 (0.08%)	0.37 (0.08–1.65)
Between 54 and 66 months (*n* = 17,193; 44.6%)		
First	19/7,999 (0.24%)	1 (ref)
Second	7/6,545 (0.11%)	0.35 (0.13–0.98)
Third	4/2,649 (0.15%)	0.65 (0.21–2.04)

Adjusted for child factors (sex, singleton/multiple births, gestational age, parity), parental factors (maternal smoking, maternal education, paternal education), and region of residence. The data are expressed as numbers, *n* (%). CI, confidence interval.

## 4. Discussion

We found that higher birth order was associated with a greater risk of hospitalization due to KD at 6–18 months of age, which coincides with the peak age of onset of KD in Japan (10). This association was not observed in patients over 18 months of age. In addition, the risk of hospitalization for KD due to higher birth order was increased for children not attending group childcare aged 6–18 months, but not for children attending group childcare.

During the COVID-19 pandemic, older children and adults were encouraged to take infection control measures, such as wearing masks in public places, maintaining social distancing, and washing hands (19, 20), owing to which many viral infections have been reduced (20, 21). Consequently, countries have reported a decrease in KD cases after the COVID-19 pandemic compared with previous years (19, 21–24). Thus, the KD incidence appears to be affected by the prevalence of infectious diseases. In contrast to older children, infants have a limited range of social activities (25, 26); therefore, infections in infants are more likely to be caused by pathogen transfer from older siblings or children of the same age living together (13, 14, 25, 26). When infections occur, neutrophils and macrophages are activated, and inflammation is elicited (8). In typical infections, inflammation is controlled and sufficient to serve as a defense system; however, in KD, inflammation is further exacerbated (3, 5, 27–29). The end result is over-activation of the immune system, leading to the development of KD (2, 4, 5, 27–29). Although KD has been studied nationwide in Japan since 1970, the relationship between the pathogenesis of KD and infectious diseases is still being investigated, and many factors associated with KD development remain unresolved. Specifically, the reason for the peak age of onset of KD being approximately one year and the risk factors associated with this age are unknown. Few reports have documented the effect of the presence of siblings on KD incidence before the age of one (15). Furthermore, to gain insight into the pathogenesis of KD, it is necessary to examine the factors that contribute to this peak age of onset through comparisons across a broader age range. In this study, we examined the association between birth order and KD incidence in children aged between 6 and 66 months, as well as the effect of group childcare on this association, using data from a nationally representative longitudinal survey of residents in Japan.

The birth order is associated with the risk of developing infectious diseases (10, 12, 14, 26). Children born later are exposed to more pathogens from their older siblings and may therefore be at a higher risk of acquiring infections. The current study supports the notion that higher birth order may increase the risk of developing KD due to the pathogens to which children are exposed during the first 6–18 months. In this study, the effect of higher birth order was enhanced in children aged 6–18 months who did not attend group childcare. For children not enrolled in group childcare, pathogen transfer is often limited to family members. Therefore, the effect of higher birth order on KD development is likely more significant. It may also suggest that infections caused by pathogens in group childcare may trigger KD, regardless of birth order. In contrast, no clear association between KD and higher birth order was observed in any age group after 18 months of age. This may be due to the increased range of social activities among older children and the increased pathogen transfer from children outside their households. This study does not imply that group childcare should be avoided, or that fewer siblings are preferable. However, this indicates that children with higher birth orders may be exposed to pathogens from their older siblings at home and that this may be involved in KD development. Further research is needed to explore these associations along with studies on the genetic and immunological susceptibility of children to KD development.

The greatest strength of this study is its use of a large, nationally representative Japanese pediatric cohort; approximately one-twentieth of all children born in Japan in 2010 were included. Therefore, the number of KD cases was relatively large. Moreover, the response rate for the baseline survey was high (88.1%), and the follow-up rate remained high at approximately 70% by the sixth survey. The incidence of KD in our cohort differed little from that reported in the Japanese National Epidemiological Survey of KD (10, 30). Thus, we believe that the cohort used in this study is appropriate for the study of children with KD in Japan. Additionally, we were able to adjust extensively for potential confounding factors, including child and parental factors, and region of residence. There have only been a few cohort studies of KD using a similarly large population. Few studies have examined the effect of the presence of siblings on KD incidence up to age one (15). Additionally, our study included children up to 66 months of age, and we observed not only the effect of birth order on KD onset but also the influence of group childcare on this relationship.

This study had several limitations. Firstly, the presence or absence of hospitalization due to KD was obtained from questionnaire responses and reported retrospectively. In addition, the data obtained from the MHLW were anonymized and direct hospitalization could not be verified. However, the diagnosis of KD is based on a set of characteristic clinical signs and symptoms. Furthermore, the Diagnostic Manual (Revision of Diagnostic Guidelines for Kawasaki Disease: 5th revised edition) (31) was used between 2002 and 2019; hence, the method used to diagnose KD should have been uniform across the country during the study period (31). In addition, children in Japan generally have good access to pediatric care because of the national health insurance system coverage, which means that most children in Japan are likely to have access to pediatric specialists. The questionnaire was answered by parental recall, and there may be some uncertainties in this regard. However, Japanese guidelines for the treatment of KD include multiple follow-up echocardiographic examinations during the first year after the onset of KD (32), allowing for easier parental recall. Therefore, the presence or absence of KD based on questionnaire responses is likely to be based on the pediatrician's diagnosis and correctly recalled by the parents and is thus considered reliable.

Secondly, this study only investigated the presence of hospitalizations due to KD; the survey made no distinction between typical, atypical, and incomplete KD. During the period covered by this study, the diagnosis of KD was made in accordance with the Diagnostic Guidelines for Kawasaki Disease (5th revised edition) (31). In the guideline, atypical KD is diagnosed when only 3–4 major symptoms are observed, and coronary artery lesions are found on echocardiography during the disease. If the patient has only 3–4 major symptoms and no coronary artery lesions, but other diseases are ruled out and KD is the most likely diagnosis based on the reference criteria, the diagnosis is incomplete KD. Furthermore, only the incidence of KD was considered in this study. Clinical information after admission, such as the presence or absence of coronary artery lesions and the assessment of coronary arteries according to *Z*-score criteria, was not available. It was not possible to examine the relationship between birth order or group childcare and KD severity. Thirdly, the study cohort was limited in birth dates (May 10–24, 2010); therefore, the seasonality of KD and infectious diseases may have affected the age of onset and the number of KD cases in the current cohort. Lastly, several studies have reported an association between KD and genetic factors, and a higher incidence of KD has been reported in siblings (33). However, in this study, we could not evaluate whether siblings were affected by KD because the questionnaires did not include this information. Further studies are needed to determine the genetic and infection-related factors involved in KD pathogenesis.

## 5. Conclusions

Using data from a nationwide longitudinal survey in Japan, we found that higher birth order was associated with KD development among children aged 6–18 months. The effect of higher birth order was more pronounced among children who did not attend group childcare. In this age group, which coincides with the peak age of onset of KD among Japanese children, exposure to infections from older siblings may be associated with the development of KD.

## Data Availability

The data analyzed in this study is subject to the following licenses/restrictions: Data supporting the results of this study are available from the Japanese Ministry of Health, Labour and Welfare. These data were used under license for this study and are not available to the public; therefore, their use is restricted. The data used in this study are available from the authors upon reasonable request and with permission from the Japanese Ministry of Health, Labour and Welfare.
