# Supplementary material for: Evaluation of the association of birth order and group childcare attendance with Kawasaki disease using data from a nationwide longitudinal survey
Source: Front Pediatr. 2023 Mar 28;11:1127053. doi: 10.3389/fped.2023.1127053 (PMC10086172; doi:10.3389/fped.2023.1127053)
Supplement: Supplementary file 1 [file Datasheet1.pdf]

㊙ 統計法に基づく一般統計調査

ひと、くらし、みらいのために
厚生労働省

第１回21世紀出生児縦断調査調査票
【平成22年出生児】

（平成22年（2010年）12月１日調査）

お答えになった内容については統計の作成以外の目的には使用しませんのでご協力をお願いします。

SAMPLE

最初に、この**調査票の回答者**についておたずねします。お子さんからみてどなたがお答えになったか、**あてはまる番号**に○をつけてください。

1　お母さん	2　お父さん	3　おばあさん	4　おじいさん	5　その他（　　　　）

問１　**どなたが保育をしているかについておたずねします。**平成22年５月に生まれたお子さんの保育は、ふだんどなたがしていますか。**あてはまる番号すべて**に○をつけてください。また、**平日の日中の主な保育者の番号ひとつを□**に記入してください。

1　お母さん	2　お父さん	3　おばあさん
4　おじいさん	5　保育所の保育士	6　保育ママさんやベビーシッター
7　その他（　　　　）		**平日の日中の主な保育者の番号** □

続いて問２→

問２　現在、平成22年５月に生まれた**お子さんはどなたと同居**していますか。**あてはまる番号すべて**に○をつけてください。お兄さん、お姉さんがいる場合は出生年月を記入してください。

※　単身赴任等で長期不在の方であっても、３か月に１度以上の割合で帰宅する場合は同居に含めます。
３か月を超えて不在の場合は同居に含めません。

1　お母さん	2　お父さん	3　お兄さん・お姉さん（　　）人	
4　お母さんの父親	5　お母さんの母親	6　お父さんの父親	7　お父さんの母親
8　お母さんの兄弟・姉妹（　　）人	9　お父さんの兄弟・姉妹（　　）人	10　その他（　　）人	

（補問２－１）　お兄さん、お姉さんの出生年月

（７人以上お子さんがいらっしゃる場合は、余白にご記入ください。）

第１子	1　男　　2　女	1　昭和　　2　平成	年	月生
第２子	1　男　　2　女	1　昭和　　2　平成	年	月生
第３子	1　男　　2　女	1　昭和　　2　平成	年	月生
第４子	1　男　　2　女	1　昭和　　2　平成	年	月生
第５子	1　男　　2　女	1　昭和　　2　平成	年	月生
第６子	1　男　　2　女	1　昭和　　2　平成	年	月生

SAMPLE

問３　現在、お母さん、お父さんは単身赴任中ですか。**それぞれあてはまる番号**に○をつけてください。

※　３か月に１度以上の割合で帰宅する場合も、３か月を超えて不在の場合も、記入してください。

**【お母さん】**	**【お父さん】**
1　はい　　2　いいえ	1　はい　　2　いいえ

続いて問４→

※ ここから、「お母さん」、「お父さん」の欄は、平成22年生まれのお子さんと同居していない場合は、空欄のままで結構です。

すべての方がお答えください

問４　出産１年前（平成21年５月）と**現在の就業状況等**についておたずねします。**あてはまる番号**に○をつけてください。「勤め(常勤)」「勤め(ﾊﾟｰﾄ･ｱﾙﾊﾞｲﾄ)」の方は、右の「企業規模・官公庁」の**あてはまる番号**に○をつけてください。

※ 単身赴任等で長期不在の方であっても、３か月に１度以上の割合で帰宅する場合は記入してください。

	【お母さん】		【お父さん】	
	**就業状況等**	**企業規模･官公庁**	**就業状況等**	**企業規模･官公庁**
出産1年前	1　無職<br>2　学生<br>3　勤め(常勤)<br>4　勤め(ﾊﾟｰﾄ･ｱﾙﾊﾞｲﾄ)<br>5　自営業･家業<br>6　内職<br>7　その他（　　　　）	（3・4の方）<br>1　1～ 4人<br>2　5～ 99人<br>3　100～499人<br>4　500人以上<br>5　官公庁	1　無職<br>2　学生<br>3　勤め(常勤)<br>4　勤め(ﾊﾟｰﾄ･ｱﾙﾊﾞｲﾄ)<br>5　自営業･家業<br>6　内職<br>7　その他（　　　　）	（3・4の方）<br>1　1～ 4人<br>2　5～ 99人<br>3　100～499人<br>4　500人以上<br>5　官公庁
現在	無職<br>1　仕事を探している<br>2　仕事を探していない<br>→ 1　近いうちに仕事をしたいと思っている<br>→ 2　当面は仕事をしたいとは思っていない<br>3　学生<br>4　勤め(常勤)<br>5　勤め(ﾊﾟｰﾄ･ｱﾙﾊﾞｲﾄ)<br>6　自営業･家業<br>7　内職<br>8　その他（　　　　）	（4・5の方）<br>1　1～ 4人<br>2　5～ 99人<br>3　100～499人<br>4　500人以上<br>5　官公庁	無職<br>1　仕事を探している<br>2　仕事を探していない<br>→ 1　近いうちに仕事をしたいと思っている<br>→ 2　当面は仕事をしたいとは思っていない<br>3　学生<br>4　勤め(常勤)<br>5　勤め(ﾊﾟｰﾄ･ｱﾙﾊﾞｲﾄ)<br>6　自営業･家業<br>7　内職<br>8　その他（　　　　）	（4・5の方）<br>1　1～ 4人<br>2　5～ 99人<br>3　100～499人<br>4　500人以上<br>5　官公庁

SAMPLE

現在、「4　勤め（常勤）」または「5　勤め（ﾊﾟｰﾄ･ｱﾙﾊﾞｲﾄ）」の方のみお答えください。

（**補問４－１**）　平成22年５月に生まれたお子さんの育児にあたって、**育児休業を取得**していますか。**あてはまる番号ひとつ**に○をつけてください。また１～３のいずれかに○をつけた方は、**取得（予定）期間**を記入してください。（産後休業（休暇）や勤務時間短縮などの部分休業は含みません。）

【お母さん】	【お父さん】
1　すでに取得した<br>2　現在、育児休業中である<br>3　これから取得する予定である<br>→ 取得（予定）期間 □ か月 □ 週<br>4　職場に育児休業制度はあるが取得しない<br>5　職場に育児休業制度がない<br>6　職場に育児休業制度があるかどうかわからない	1　すでに取得した<br>2　現在、育児休業中である<br>3　これから取得する予定である<br>→ 取得（予定）期間 □ か月 □ 週<br>4　職場に育児休業制度はあるが取得しない<br>5　職場に育児休業制度がない<br>6　職場に育児休業制度があるかどうかわからない

（**補問４－２**）

取得しない理由をひとつ選んで番号に○をつけてください。

【お母さん】	【お父さん】
1　職場の雰囲気や仕事の状況から	1　職場の雰囲気や仕事の状況から
2　経済的なことから	2　経済的なことから
3　仕事に早く復帰したいから	3　仕事を続けたいから
4　夫が育児休業をとっているから	4　妻が育児休業をとっているから
5　その他（　　　　　　　　　）	5　その他（　　　　　　　　　）

SAMPLE

（補問４－３）　**現在、出産１年前**（平成21年５月）と**比べて、働き方に変わったこと**がありますか。**あてはまる番号すべて**に○をつけてください。

【お母さん】	【お父さん】
1　出産・育児のため職場内で仕事が変わった	1　育児のため職場内で仕事が変わった
2　始業・終業時刻の繰上げ、繰下げを行った	2　始業・終業時刻の繰上げ、繰下げを行った
3　短時間勤務をするようになった	3　短時間勤務をするようになった
4　フレックスタイム制度を利用した	4　フレックスタイム制度を利用した
5　在宅勤務制度を利用した	5　在宅勤務制度を利用した
6　残業をしなくなったまたは減らした	6　残業をしなくなったまたは減らした
7　残業が増えた	7　残業が増えた
8　変わらない	8　変わらない

（補問４－４）　**現在、出産１年前**（平成21年５月）と**比べて、生活スタイルに変わったこと**がありますか。**あてはまる番号すべて**に○をつけてください。

【お母さん】	【お父さん】
1　家事・育児の従事時間が増えた（育児休業中を含む）	1　家事・育児の従事時間が増えた（育児休業中を含む）
2　仕事以外の交際時間等を減らした	2　仕事以外の交際時間等を減らした
3　変わらない	3　変わらない

SAMPLE

出産１年前（平成21年５月）の仕事をやめられた方のみお答えください。

（補問４－５）　**出産１年前（平成21年５月）の仕事をやめた理由にあてはまる番号すべて**に○をつけてください。また、その中で**一番の理由と思われるものの番号**を番号記入欄に記入してください。

【お母さん】	【お父さん】
1　育児に専念したいため、自発的にやめた	1　育児に専念したいため、自発的にやめた
2　仕事を続けたかったが、両立が難しいのでやめた	2　仕事を続けたかったが、両立が難しいのでやめた
3　解雇された、退職勧奨された	3　解雇された、退職勧奨された
4　出産・育児に関係なく自発的にやめた	4　出産・育児に関係なく自発的にやめた
5　妊娠に関連した健康上の理由でやめた	5　健康上の理由でやめた
6　その他　（　　　　　　　　　　　）	6　その他　（　　　　　　　　　　　）
7　特に理由はない	7　特に理由はない
**一番の理由と思われるものの番号記入欄** □	**一番の理由と思われるものの番号記入欄** □

SAMPLE

すべての方がお答えください。

問５　最近１週間の家事・育児以外の労働時間についておたずねします。**あてはまる番号**に○をつけてください。

（補問５－１）　**労働時間がある方の片道の通勤時間**
あてはまる番号に○をつけてください。

【お母さん】

1　なし
2　20時間未満
3　20時間以上40時間未満
4　40時間以上50時間未満
5　50時間以上60時間未満
6　60時間以上

（通勤時間は含みません。）

→（2～6の方）
1　通勤に時間はかからない
2　30分未満
3　30分以上１時間未満
4　１時間以上１時間30分未満
5　１時間30分以上

【お父さん】

1　なし
2　20時間未満
3　20時間以上40時間未満
4　40時間以上50時間未満
5　50時間以上60時間未満
6　60時間以上

（通勤時間は含みません。）

→（2～6の方）
1　通勤に時間はかからない
2　30分未満
3　30分以上１時間未満
4　１時間以上１時間30分未満
5　１時間30分以上

問６　**育児や家事の分担状況**についておたずねします。①～⑥のそれぞれについて、**あてはまる番号にひとつずつ**○をつけてください。

		【お母さん】いつもする	【お母さん】ときどきする	【お母さん】ほとんどしない	【お母さん】まったくしない	【お父さん】いつもする	【お父さん】ときどきする	【お父さん】ほとんどしない	【お父さん】まったくしない
育児	①　食事の世話をする	1	2	3	4	1	2	3	4
育児	②　おむつを取り換える	1	2	3	4	1	2	3	4
育児	③　入浴させる	1	2	3	4	1	2	3	4
育児	④　寝かしつける	1	2	3	4	1	2	3	4
育児	⑤　家の中で相手をする	1	2	3	4	1	2	3	4
育児	⑥　散歩など屋外に連れて行く	1	2	3	4	1	2	3	4
家事	①　食事をつくる	1	2	3	4	1	2	3	4
家事	②　食事の後片づけをする	1	2	3	4	1	2	3	4
家事	③　部屋等の掃除をする	1	2	3	4	1	2	3	4
家事	④　洗濯をする	1	2	3	4	1	2	3	4
家事	⑤　ゴミを出す	1	2	3	4	1	2	3	4
家事	⑥　日常の買い物をする	1	2	3	4	1	2	3	4

SAMPLE

問７　平成22年５月に生まれたお子さんについて、**保育所、家庭的保育（保育ママ）等の保育サービス（一時的なサービスを除く）を利用（入所）**していますか。**あてはまる番号ひとつ**に○をつけてください。

１　利用している　　　２　利用していない

（補問７－１）

「１　利用している」場合、利用（入所）している保育サービスに**あてはまる番号すべて**に○をつけてください。

１　認可保育所（公立）
２　認可保育所（私立）
３　認定こども園
４　自治体独自の保育施設（認証保育所など）
５　事業所内（企業内）保育施設
６　認可外保育施設（事業所内（企業内）保育施設をのぞく）
７　家庭的保育（保育ママ）
８　ベビーシッター
９　その他（　　　　　　　　）

（補問７－２）

利用している保育サービスは、希望どおりのものですか。**あてはまる番号ひとつ**に○をつけてください。

１　はい　　　２　いいえ

（補問７－３）

「２　いいえ」の場合、利用したかった保育サービスを、**補問７－１の選択肢の中からひとつ選び、□に番号**を記入してください。

利用をしたかった保育サービス　□

（補問７－４）

「２　利用していない」場合、利用していない理由は何ですか。**あてはまる番号ひとつ**に○をつけてください。

１　必要がない
２　利用したい保育サービスに空きがない
３　利用したい保育サービスがない
４　利用したい保育サービスはあるが、経済的理由により利用できない
５　その他（　　　　　　　　）

（補問７－５）

２～４に○をつけた場合、利用したい保育サービス（一時的なサービスを除く）は何ですか。**あてはまる番号すべて**に○をつけてください。

１　認可保育所（公立）
２　認可保育所（私立）
３　認定こども園
４　自治体独自の保育施設（認証保育所など）
５　事業所内（企業内）保育施設
６　認可外保育施設（事業所内（企業内）保育施設をのぞく）
７　家庭的保育（保育ママ）
８　ベビーシッター
９　その他（　　　　　　　　）

SAMPLE

問８　たばこを吸っていますか。あてはまる番号に○をつけてください。「吸っている」と答えた方は家庭ではどうかの補問にお答えください。

【お母さん】	【お父さん】
1　吸っていない<br>2　吸っている ──→ 1日 [　　] 本<br>└→（補問）　1　室内で吸う<br>2　室内では吸わない	1　吸っていない<br>2　吸っている ──→ 1日 [　　] 本<br>└→（補問）　1　室内で吸う<br>2　室内では吸わない

問９　日ごろ、子育てで意識して行っていることは何ですか。あてはまる番号すべてに○をつけてください。

1　よく話しかける
2　よくだっこする
3　よい音楽をきかせる
4　外気浴をさせる
5　子どもの生活リズムをくずさない
6　その他（　　　　　　　　　　　　　　　　　）
7　特に意識して行っていることはない

SAMPLE

問10　平成22年５月に生まれたお子さんをもってよかったと思うことは何ですか。あてはまる番号すべてに○をつけてください。

1　家庭が明るくなった
2　身近な人が喜んでくれた
3　生活にはりあいができた
4　上の子に弟・妹ができた
5　子育てを通じて自分の友人が増えた
6　子育てを通じて自分の視野が広がった
7　その他（　　　　　　　　　　　　　　　　　）
8　よかったと思うことは特にない

問11　平成22年５月に生まれたお子さんをもって負担に思うことは何ですか。あてはまる番号すべてに○をつけてください。

1　子育てによる身体の疲れが大きい
2　子育てで出費がかさむ
3　自分の自由な時間が持てない
4　夫婦で楽しむ時間がない
5　仕事が十分にできない
6　子育てが大変なことを身近な人が理解してくれない
7　子どもが病気がちである
8　その他（　　　　　　　　　　　　　　　　　　）
9　負担に思うことは特にない

問12　子育ての不安や悩みがありますか。あてはまる番号ひとつに○をつけてください。

1　すごくある
2　少しある
3　ほとんどない

（補問12－１）そのことで相談する場合は、誰に相談しますか。
あてはまる番号すべてに○をつけてください。「ほとんどない」方もお答えください。

1　配偶者	2　自分の両親	3　配偶者の両親
4　親戚	5　友人・知人	6　医師
7　保健師	8　助産師・看護師	9　保育士
10　カウンセラー（電話相談を含む）		11　育児サークルの仲間
12　その他（　　　　　　　　　　　）		
13　誰にも相談しない		

SAMPLE

問13　子育ての不安や負担を解消するために利用したいサービスはありますか。あてはまる番号すべてに○をつけてください。

1　一時的に自宅以外で子供を預かってくれるサービス（一時預かりサービス）
2　一時的に自宅で子どもを見てくれるサービス（ベビーシッターなど）
3　親子ひろば、子育てひろばなどの地域子育て支援拠点
4　育児相談や支援のために自宅に訪問してくれるサービス
5　その他（　　　　　　　　　　　　　　　　　）
6　特にない

問14　平成22年５月に生まれたお子さんに、**母乳、人工乳（粉ミルク）を何か月間与えましたか。**
あてはまる番号に○をつけ、**与えた期間**を記入してください。混合して与えた場合はそれぞれに記入してください。

**母乳**	1　与えた（与えている）→ □か月<br>2　与えなかった<br>3　初乳のみ与えた	**人工乳**	1　与えた（与えている）→ □か月<br>2　与えなかった

問15　**平成21年１年間のお母さん、お父さんの年収（税込み）およびその他の年間収入**についておたずねします。それぞれ**あてはまる番号**に○をつけ、**□には金額**を記入してください。分けられない場合は、どちらかにまとめて記入していただいて結構です。１万円未満は四捨五入してください。

※ 単身赴任等で長期不在の方の分についても、記入してください。

平成21年の年収	お母さんの働いて得た年収	お父さんの働いて得た年収	その他の年間収入（親からの援助、家賃・地代等の財産収入、児童手当・出産一時金等社会保障給付金等を含みます。）
	1　あり □万円<br>2　なし	1　あり □万円<br>2　なし	1　あり □万円<br>2　なし

問16　**平成22年11月中の養育費**についておたずねします。**平成22年５月に生まれたお子さんにかかった子育て費用**を記入してください。子育て費用とは、人工乳などの食費、紙おむつ代、衣類、保育料、絵本、おもちゃ代などの合計です。

子育て費用　□万円

そのうち、保育料（保育所や保育ママさんなどに支払った費用）があれば記入してください。

保育料　□万 □千円

問17　**平成22年５月に生まれたお子さんの子ども手当について、どのように感じていますか。**
あてはまる番号ひとつに○をつけてください。

1　非常に役に立つ
2　役に立つ
3　どちらともいえない
4　あまり役に立たない
5　役に立たない

SAMPLE

※　最後に、お子さんを育てておられてふだん感じていること、この調査に関することなど何でも結構ですので、自由にご記入ください。
なお、下記の記載欄に記入された内容について、個人が特定できないようにした上で、お便りや白書などでご紹介させていただくことがありますので、使用してもよろしければ□欄にチェックをお願いします。

使用可 □

SAMPLE

ご協力ありがとうございました。

最後にもう一度、記入されていないページがないかご確認のうえ、お早めに、同封の返送用封筒にてご投函ください。
なお、この調査の結果は、まとまり次第皆様のもとにお届けします。
今後ともご協力をいただきますようお願いします。

※ この調査についての連絡・問い合わせ先

厚生労働省 大臣官房 統計情報部
社会統計課 縦断調査室 B

代表電話 (03) 5253-1111(内線7566)
直通電話 (03) 3595-2321
Eメール b-cohort@mhlw.go.jp

電話による問い合わせは、
平日の月曜～金曜の午前９時30分から
午後６時15分までにお願いします。
