# Supplementary material for: Evaluation of the association of birth order and group childcare attendance with Kawasaki disease using data from a nationwide longitudinal survey
Source: Front Pediatr. 2023 Mar 28;11:1127053. doi: 10.3389/fped.2023.1127053 (PMC10086172; doi:10.3389/fped.2023.1127053)
Supplement: Supplementary file 2 [file Datasheet2.pdf]

㊙ 統計法に基づく一般統計調査

第２回21世紀出生児縦断調査調査票【平成22年出生児】

（平成23年(2011年)12月１日調査）

ひと、くらし、みらいのために
厚生労働省

お答えになった内容については統計の作成以外の目的には使用しませんのでご協力をお願いします。

最初に、この**調査票の回答者**についておたずねします。平成22年５月生まれのお子さんからみてどなたがお答えになったか、**あてはまる番号**に○をつけてください。

1　お母さん	3　お母さんの母親	5　お父さんの母親	7　その他(具体的に　　　)
2　お父さん	4　お母さんの父親	6　お父さんの父親	

【ご家族について】

問１　現在、平成22年５月生まれのお子さんはどなたと同居していますか。**あてはまる番号すべて**に○をつけ、□には**人数**を記入してください。（03の兄弟姉妹の人数には、この調査票の対象となっている平成22年５月生まれのお子さんは含めません。また、平成22年５月生まれのお子さんが双子、三つ子の場合は、他の調査票の対象となっているお子さんも人数に含めてください。）

※　単身赴任等で長期不在の方であっても、３か月に１度以上の割合で帰宅する場合は同居に含めます。３か月を超えて不在の場合は同居に含めません。

01　お母さん	04　お母さんの母親	08　お母さんの兄弟・姉妹　□人
02　お父さん	05　お母さんの父親	09　お父さんの兄弟・姉妹　□人
03　兄弟姉妹（本人を除く）□人	06　お父さんの母親	10　その他(具体的に　　　)　□人
	07　お父さんの父親	

SAMPLE

（補問１－１）　前回の調査時（平成22年(2010年)12月１日）以降、同居の**兄弟姉妹が増えた場合**、そのお子さんの性別、出生年の元号に○をつけ、□に**出生年月**を記入してください。

1　男　2　女	1　昭和　2　平成	□年	□月生
1　男　2　女	1　昭和　2　平成	□年	□月生
1　男　2　女	1　昭和　2　平成	□年	□月生

問２　平成22年５月生まれのお子さんのふだんの保育はどなたがしていますか。**あてはまる番号すべて**に○をつけてください。

1　お母さん	4　お母さんの父親	7　保育所・託児所の保育士
2　お父さん	5　お父さんの母親	8　保育ママさんやベビーシッター
3　お母さんの母親	6　お父さんの父親	9　その他(具体的に　　　)

（補問２－１）　そのうち、**平日の日中**にお子さんと一緒にいる時間が**一番長い**のはどなたですか。**あてはまる番号ひとつを上の欄の中から選んで**□にその番号を記入してください。

平日の日中の主な保育者　□

問３　現在、お母さん、お父さんは**単身赴任中**ですか。**それぞれあてはまる番号**に○をつけてください。
※　３か月に１度以上の割合で帰宅する場合も、３か月を超えて不在の場合も、記入してください。

【お母さん】	【お父さん】
１　はい　　２　いいえ	１　はい　　２　いいえ

問４　平成22年５月生まれのお子さんが、おばあさん、おじいさんとこれまで**行き来した回数**はどの程度ですか。**あてはまる番号ひとつ**に○をつけてください。

【お母さんの母親】	【お母さんの父親】	【お父さんの母親】	【お父さんの父親】
１　同居している	１　同居している	１　同居している	１　同居している
〔別居している〕	〔別居している〕	〔別居している〕	〔別居している〕
２　ほとんど毎日	２　ほとんど毎日	２　ほとんど毎日	２　ほとんど毎日
３　週に２～３回程度	３　週に２～３回程度	３　週に２～３回程度	３　週に２～３回程度
４　月に２～３回程度	４　月に２～３回程度	４　月に２～３回程度	４　月に２～３回程度
５　月に１回程度	５　月に１回程度	５　月に１回程度	５　月に１回程度
６　数回	６　数回	６　数回	６　数回
７　行き来しなかった	７　行き来しなかった	７　行き来しなかった	７　行き来しなかった
８　いない	８　いない	８　いない	８　いない

【平成22年５月生まれのお子さんについて】

問５　平成22年５月生まれのお子さんの**食事で気をつけていること**についておたずねします。**あてはまる番号すべて**に○をつけてください。

01　いろいろな種類の食品を食べさせるようにしている
02　子どもが好きなものを食べさせるようにしている
03　子どもが嫌いなものでも食べさせるようにしている
04　多くの量を食べさせるようにしている
05　子どもの健康や成長によくないといわれるものは食べさせないようにしている
06　決まった時間に食べさせるようにしている
07　子どもが欲しがる時に食べさせるようにしている
08　家族がそろった中で食べさせるようにしている
09　その他（具体的に　　　　　　　　　　）
10　気をつけていることは特にない

SAMPLE

問６　平成22年５月生まれのお子さんがふだん**何時ごろに寝るか**おたずねします。**あてはまる番号ひとつ**に○をつけてください。

１　午後７時前	５　午後10時台
２　午後７時台	６　午後11時以降
３　午後８時台	７　不規則である
４　午後９時台	

問７　平成22年５月生まれのお子さんの**遊び**のようすについておたずねします。

（1）**誰と一緒に**遊んでいますか。**あてはまる番号すべてに**○をつけてください。（休日に遊ぶ人も含めてください。）

01　兄弟姉妹	05　同い年ぐらいの子ども	09　一人遊びが多い
02　お母さん	06　年上の子ども	10　その他（具体的に　　　　）
03　お父さん	07　親せきや近所のおとな	11　わからない
04　お母さん・お父さんの親	08　保育士や保育ママさん、ベビーシッター	

（2）**どんな遊び**が多いですか。**主な番号３つまで**に○をつけてください。

01　積み木・ブロック	06　ビデオ・ＤＶＤ・テレビ	11　三輪車などの乗り物
02　人形・ぬいぐるみ	07　歌・踊り	12　お散歩
03　ままごと	08　砂遊び・水遊び	13　子ども向けのプレイルーム
04　お絵かき	09　すべり台など戸外の遊具	14　その他（具体的に　　　　）
05　絵本・お話	10　ボール遊び	15　わからない

問８　平成22年５月生まれのお子さんは、この**１年の間**（平成22年12月から平成23年11月まで）**に病院や診療所などで診察を受けた病気やけが**がありましたか。**あてはまる番号すべて**に○をつけてください。

01　病院や診療所などで診察を受けるほどの病気やけがはなかった ──→ **問９へ**

02　百日ぜき	13　結膜炎（アレルギー性は09へ）	22　けいれん、ひきつけ
03　水痘〔水ぼうそう〕	14　中耳炎、外耳炎	23　発達と行動面の相談
04　麻疹〔はしか〕	15　かぜ、咽頭炎、扁桃(腺)炎、気管支炎、肺炎	24　う歯〔むし歯〕
05　風しん〔三日はしか〕	16　インフルエンザ	25　その他の病気（具体的に　　　　）
06　突発性発疹	17　胃腸炎など消化器系の病気、下痢、腹痛、便秘などの症状（腸重積は07へ）	26　打撲、切り傷
07　腸重積	18　伝染性膿痂疹〔とびひ〕	27　骨折
08　川崎病	19　湿疹（アトピー性皮膚炎は11へ）	28　やけど
09　アレルギー性鼻炎、アレルギー性結膜炎	20　その他の皮膚炎	29　その他のけが（病気以外の原因によるもの）（具体的に　　　　）
10　ぜんそく	21　先天性の病気	
11　アトピー性皮膚炎		
12　食物アレルギー		

（補問８－１）　○をつけた番号のうち、入院した（している）病気やけががある場合には、その番号を□に記入してください。（６つ以上ある場合は、余白に記入してください。）

入院した（している）病気やけが　□　□　□　□　□

SAMPLE

問９　平成22年５月生まれのお子さんは、この**１年の間**（平成22年12月から平成23年11月まで）に次のようなことがありましたか。**あてはまる番号すべて**に○をつけてください。（病院や診療所などで診察を受けなかった場合も含めてください。）

01　ベッドや階段、いすなどから転落した
02　ドア、窓などに手足などをはさまれた
03　刃物やガラス片など鋭利なものに触れ、手足などを切った
04　動物にかまれた、ハチなどに刺された
05　浴そうや池などでおぼれた、おぼれそうになった
06　コイン、化粧品、洗剤、たばこなどを誤って飲んだ
07　目・耳・鼻に異物が入った
08　アイロンや熱い鍋などに接触したり、熱湯を浴びた
09　交通事故にあった
10　その他の事故等（具体的に　　　　　　　　　　）
11　特になかった

問10　平成22年５月生まれのお子さんの**現在の身長・体重、測定した日**を□に記入してください。母子健康手帳の「１歳６か月健康診査」の記録を見て記入していただいても結構です。

身　長　□□□．□ cm　　**体　重**　□□．□ kg　　**測定した日**　平成□□年□□月□□日

（年を西暦で記入する場合は下２桁を記入してください。）

SAMPLE

問11　**平成22年５月生まれのお子さんについて**次のことをおたずねします。**「はい」「いいえ」のいずれかの番号**に○をつけてください。

①　ひとりで上手に歩くことができる	1　はい	2　いいえ
②「ママ」、「ブーブー」など意味のある言葉を言う	1　はい	2　いいえ
③　自分でコップを持って水を飲むことができる	1　はい	2　いいえ
④　ほ乳ビンを使っている	1　はい	2　いいえ
⑤　食事やおやつの時間はだいたい決まっている	1　はい	2　いいえ
⑥　保護者が歯の仕上げみがきをしてあげている	1　はい	2　いいえ
⑦　極端にまぶしがったり、目の動きがおかしいのではないかと気になる	1　はい	2　いいえ
⑧　うしろから名前を呼んだとき、振り向く	1　はい	2　いいえ
⑨　絵本をめくったり、クレヨンなどでなぐり描きをする	1　はい	2　いいえ
⑩　他の子どものしぐさや行動をまねたり、同じおもちゃを欲しがる	1　はい	2　いいえ

【お母さん・お父さんについて】

問12　お母さん、お父さんの**食習慣**についておたずねします。**「はい」「いいえ」のいずれかの番号**に○をつけてください。

※　単身赴任等で長期不在の方であっても、３か月に１度以上の割合で帰宅する場合は記入してください。

【お母さん】

①　１日３回の食事をとるようにしている	1　はい	2　いいえ
②　夜食や間食をすることが多い	1　はい	2　いいえ
③　朝食はとるようにしている	1　はい	2　いいえ
④　食事は決まった時間にとるようにしている	1　はい	2　いいえ
⑤　いろいろな種類の食品を食べるようにしている	1　はい	2　いいえ
⑥　塩分のとり過ぎに気をつけている	1　はい	2　いいえ
⑦　糖分のとり過ぎに気をつけている	1　はい	2　いいえ
⑧　カロリーのとり過ぎに気をつけている	1　はい	2　いいえ

【お父さん】

①　１日３回の食事をとるようにしている	1　はい	2　いいえ
②　夜食や間食をすることが多い	1　はい	2　いいえ
③　朝食はとるようにしている	1　はい	2　いいえ
④　食事は決まった時間にとるようにしている	1　はい	2　いいえ
⑤　いろいろな種類の食品を食べるようにしている	1　はい	2　いいえ
⑥　塩分のとり過ぎに気をつけている	1　はい	2　いいえ
⑦　糖分のとり過ぎに気をつけている	1　はい	2　いいえ
⑧　カロリーのとり過ぎに気をつけている	1　はい	2　いいえ

SAMPLE

問13　お母さん、お父さんが最後に**卒業した**（あるいは在学中の）**学校**についておたずねします。**あてはまる番号ひとつ**に○をつけてください。

※　単身赴任等で長期不在の方の分についても、記入してください。

【お母さん】	【お父さん】
1　中学校	1　中学校
2　専修・専門学校（中学校卒業後）	2　専修・専門学校（中学校卒業後）
3　高校	3　高校
4　専修・専門学校（高校卒業後）	4　専修・専門学校（高校卒業後）
5　短大・高専	5　短大・高専
6　大学	6　大学
7　大学院	7　大学院
8　その他（具体的に　　　　）	8　その他（具体的に　　　　）

問14　平成23年12月１日現在の**就業状況**についておたずねします。**あてはまる番号**に○をつけてください。01、02に○をした方は09または10のいずれかにも○をしてください。10に○をした方は11または12のいずれかにも○をしてください。

※　単身赴任等で長期不在の方であっても、３か月に１度以上の割合で帰宅する場合は記入してください。
※　現在、育児休業などで休業中の方は、復職するときの仕事に○をつけてください。

【お母さん】	【お父さん】
01　家事(専業)<br>02　無職<br>→ 09　仕事を探している<br>→ 10　探していない<br>→ 11　近いうちに仕事をしたいと思っている<br>→ 12　当面は仕事をしたいとは思っていない	01　家事(専業)<br>02　無職<br>→ 09　仕事を探している<br>→ 10　探していない<br>→ 11　近いうちに仕事をしたいと思っている<br>→ 12　当面は仕事をしたいとは思っていない
03　学生	03　学生
04　勤め(常勤)	04　勤め(常勤)
05　勤め(パート・アルバイト)	05　勤め(パート・アルバイト)
06　自営業・家業	06　自営業・家業
07　内職	07　内職
08　その他(具体的に　　　)	08　その他(具体的に　　　)

SAMPLE

問15　**ふだんの１週間の家事・育児以外の労働時間**についておたずねします。**あてはまる番号ひとつ**に○をつけてください。

※　単身赴任等で長期不在の方であっても、３か月に１度以上の割合で帰宅する場合は記入してください。
※　通勤時間、食事時間、休憩時間は労働時間に含めないでください。
※　現在、育児休業などで休業中の方は、「１　なし」に○をつけてください。

【お母さん】	【お父さん】
1　なし	1　なし
2　20時間未満	2　20時間未満
3　20時間～40時間未満	3　20時間～40時間未満
4　40時間～50時間未満	4　40時間～50時間未満
5　50時間～60時間未満	5　50時間～60時間未満
6　60時間以上	6　60時間以上

現在、「04 勤め(常勤)」または「05 勤め(パート・アルバイト)」の方のみお答えください。

問16 平成22年５月生まれのお子さんの育児にあたって、**この１年の間**（平成22年12月から平成23年11月まで）**に職場で利用した制度**についておたずねします。**あてはまる番号**に○をつけてください。**3**に○をした方は**4**または**5**のいずれかにも○をしてください。

※ 単身赴任等で長期不在の方であっても、３か月に１度以上の割合で帰宅する場合は記入してください。

	【お母さん】この１年の間の利用状況：利用した（している）	【お母さん】この１年の間の利用状況：職場に制度はあるが利用しなかった（していない）	【お母さん】この１年の間の利用状況：職場に制度がない・わからない	【お母さん】利用希望：職場に制度があれば利用したいと思う	【お母さん】利用希望：職場に制度があっても利用したいと思わない	【お父さん】この１年の間の利用状況：利用した（している）	【お父さん】この１年の間の利用状況：職場に制度はあるが利用しなかった（していない）	【お父さん】この１年の間の利用状況：職場に制度がない・わからない	【お父さん】利用希望：職場に制度があれば利用したいと思う	【お父さん】利用希望：職場に制度があっても利用したいと思わない
① 育児休業制度	1	2	3	4	5	1	2	3	4	5
② 短時間勤務制度	1	2	3	4	5	1	2	3	4	5
③ 在宅勤務制度	1	2	3	4	5	1	2	3	4	5
④ 深夜業の免除	1	2	3	4	5	1	2	3	4	5
⑤ 時間外労働の制限または免除する制度	1	2	3	4	5	1	2	3	4	5
⑥ フレックスタイム制度	1	2	3	4	5	1	2	3	4	5
⑦ 始業・終業時刻の繰上げ・繰下げ	1	2	3	4	5	1	2	3	4	5
⑧ 事業所内（企業内）保育施設	1	2	3	4	5	1	2	3	4	5
⑨ 再雇用制度	1	2	3	4	5	1	2	3	4	5
⑩ 子の看護休暇	1	2	3	4	5	1	2	3	4	5
⑪ その他の仕事と家庭の両立支援に関する制度（具体的に　　　　　　）	1	2	3	4	5	1	2	3	4	5

SAMPLE

すべての方がお答えください。

問17　**育児や家事の分担状況**についておたずねします。育児（①～⑥）、家事（①～⑥）のそれぞれについて、**あてはまる番号にひとつずつ○**をつけてください。

※　単身赴任等で長期不在の方であっても、3か月に1度以上の割合で帰宅する場合は記入してください。

		【お母さん】いつもする	【お母さん】ときどきする	【お母さん】ほとんどしない	【お母さん】まったくしない	【お父さん】いつもする	【お父さん】ときどきする	【お父さん】ほとんどしない	【お父さん】まったくしない
育児	①　食事の世話をする	1	2	3	4	1	2	3	4
	②　おむつを取り換える	1	2	3	4	1	2	3	4
	③　入浴させる	1	2	3	4	1	2	3	4
	④　寝かしつける	1	2	3	4	1	2	3	4
	⑤　家の中で話し相手や遊び相手をする	1	2	3	4	1	2	3	4
	⑥　屋外へ遊びに連れていく	1	2	3	4	1	2	3	4
家事	①　食事をつくる	1	2	3	4	1	2	3	4
	②　食事の後片づけをする	1	2	3	4	1	2	3	4
	③　部屋等の掃除をする	1	2	3	4	1	2	3	4
	④　洗濯をする	1	2	3	4	1	2	3	4
	⑤　ゴミを出す	1	2	3	4	1	2	3	4
	⑥　日常の買い物をする	1	2	3	4	1	2	3	4

SAMPLE

問18　平成22年5月生まれのお子さんの相手をしたり、食事をしたりして**一緒に過ごしている時間**は、1日平均どのくらいですか。**あてはまる番号ひとつ**に○をつけてください。（お子さんが眠っている時間は除いてください。）

※　単身赴任等で長期不在の方であっても、3か月に1度以上の割合で帰宅する場合は記入してください。
※　「休日」については、お母さん、お父さんそれぞれの休日を記入してください。また、「家事（専業）」「無職」の方はご家族の休みの日を休日として記入してください。

【お母さん】平　日	【お母さん】休　日	【お父さん】平　日	【お父さん】休　日
1　な　し	1　な　し	1　な　し	1　な　し
2　30分未満	2　2時間未満	2　30分未満	2　2時間未満
3　30分～1時間未満	3　2時間～4時間未満	3　30分～1時間未満	3　2時間～4時間未満
4　1時間～2時間未満	4　4時間～6時間未満	4　1時間～2時間未満	4　4時間～6時間未満
5　2時間～4時間未満	5　6時間～8時間未満	5　2時間～4時間未満	5　6時間～8時間未満
6　4時間～6時間未満	6　8時間～10時間未満	6　4時間～6時間未満	6　8時間～10時間未満
7　6時間以上	7　10時間以上	7　6時間以上	7　10時間以上

【保育サービスについて】

問19 平成22年５月生まれのお子さんについて、**保育所、家庭的保育（保育ママ）等の保育サービス（一時的なサービスを除く）を利用（入所）**していますか。**あてはまる番号ひとつ**に○をつけてください。

1　利用している	2　利用していない

（補問19－１）
「１　利用している」場合、利用（入所）している保育サービスに**あてはまる番号すべて**に○をつけてください。

1　認可保育所（公立）
2　認可保育所（私立）
3　認定こども園
4　自治体独自の保育施設（認証保育所など）
5　事業所内（企業内）保育施設
6　認可外保育施設（事業所内（企業内）保育施設をのぞく）
7　家庭的保育（保育ママ）
8　ベビーシッター
9　その他（具体的に　　　　　　　　　）

（補問19－２）
ふだんの保育サービスの利用日数・時間はどのくらいですか。□には**１週間の利用日数**を記入し、**平日１日平均の利用時間にあてはまる番号ひとつ**に○をつけてください。

１週間の利用日数　週 □ 日

平日１日平均の利用時間
1　７時間未満
2　７時間～８時間未満
3　８時間～９時間未満
4　９時間～10時間未満
5　10時間～11時間未満
6　11時間～12時間未満
7　12時間以上

（補問19－３）
利用している保育サービスの種類は、希望どおりのものですか。**あてはまる番号ひとつ**に○をつけてください。

1　はい	2　いいえ

（補問19－４）
「２　いいえ」の場合、利用したかった保育サービスを、**補問19－１の太枠内１～９の中からひとつ選び**、□に**番号**を記入してください。

利用したかった保育サービス □

（補問19－５）
「２　利用していない」場合、利用していない理由は何ですか。**あてはまる番号ひとつ**に○をつけてください。

1　必要がない
2　利用したい保育サービスに空きがない
3　利用したい保育サービスはあるが、居住地域にない
4　利用したい保育サービスはあるが、経済的理由により利用できない
5　その他（具体的に　　　　　　　　　）

（補問19－６）
２～４に○をつけた場合、利用したい保育サービス（一時的なサービスを除く）は何ですか。**あてはまる番号すべて**に○をつけてください。

1　認可保育所（公立）
2　認可保育所（私立）
3　認定こども園
4　自治体独自の保育施設(認証保育所など)
5　事業所内（企業内）保育施設
6　認可外保育施設（事業所内（企業内）保育施設をのぞく）
7　家庭的保育（保育ママ）
8　ベビーシッター
9　その他（具体的に　　　　　　　　　）

SAMPLE

【子育てについて】

問20　平成22年５月生まれのお子さんを育てていて**よかったと思うこと**は何ですか。**あてはまる番号すべて**に○をつけてください。

1　家族の結びつきが深まった
2　子どもとのふれあいが楽しい
3　毎日の生活にはりあいができた
4　上の子に、兄・姉の自覚がめばえた
5　子育てを通じて自分の友人が増えた
6　子育てを通じて自分の視野が広がった
7　その他（具体的に　　　　　　　　　　　　　）
8　よかったと思うことは特にない

問21　平成22年５月生まれのお子さんを育てていて**負担に思うこと**は何ですか。**あてはまる番号すべて**に○をつけてください。

01　子育てによる身体の疲れが大きい
02　子育てで出費がかさむ
03　自分の自由な時間が持てない
04　夫婦で楽しむ時間がない
05　仕事が十分にできない
06　子育てが大変なことを身近な人が理解してくれない
07　子どもが病気がちである
08　目が離せないので気が休まらない
09　その他（具体的に　　　　　　　　　　　　　）
10　負担に思うことは特にない

SAMPLE

問22　平成22年５月生まれのお子さんを育てていて**不安や悩み**がありますか。**あてはまる番号ひとつ**に○をつけてください。

1　すごくある　　　2　少しある　　　3　ほとんどない

（補問22－１）　そのことで相談する場合は、**誰に相談**しますか。**あてはまる番号すべて**に○をつけてください。「ほとんどない」方もお答えください。

01　配偶者	06　その他の親せき	11　保育士
02　自分の親	07　友人・知人	12　カウンセラー（電話相談を含む）
03　配偶者の親	08　医師	13　育児サークルの仲間
04　自分の兄弟姉妹	09　保健師	14　その他（具体的に　　　　　）
05　配偶者の兄弟姉妹	10　助産師・看護師	15　誰にも相談しない

問23　子育ての不安や負担を解消するために利用したいサービスはありますか。あてはまる番号すべてに○をつけてください。

1　一時的に自宅以外で子どもを預かってくれるサービス（一時預かりサービス）
2　一時的に自宅で子どもを見てくれるサービス（ベビーシッターなど）
3　親子ひろば、子育てひろばなどの地域子育て支援拠点
4　育児相談や支援のために自宅に訪問してくれるサービス
5　その他（具体的に　　　　　　　　　　　　）
6　特にない

【収入、子育て費用について】

問24　平成22年１年間のお母さん、お父さんの年収（税込み）およびその他の年間収入についておたずねします。**それぞれあてはまる番号**に○をつけ、**□には金額**を記入してください。分けられない場合は、どちらかにまとめて記入していただいて結構です。

　１万円未満は四捨五入してください。

※　単身赴任等で長期不在の方の分についても、記入してください。

	お母さんの働いて得た年収	お父さんの働いて得た年収	その他の年間収入（親からの援助、家賃・地代等の財産収入、子ども手当・出産一時金等社会保障給付金等を含みます。）
平成22年の年収	1　あり □ 万円<br>2　なし	1　あり □ 万円<br>2　なし	1　あり □ 万円<br>2　なし

SAMPLE

問25　平成23年11月中の子育て費用についておたずねします。**平成22年５月生まれのお子さんにかかった費用**（紙おむつ代、衣類、保育料、絵本、おもちゃ代、おやつ代など）を□に記入してください。

※　ご家族共同で利用するものの購入や住宅の増改築の費用は含めないでください。

**11月中に平成22年５月生まれのお子さんにかかった費用**	□ 万円

※　１万円未満は四捨五入してください。
　５千円未満または子育て費用の支出がない場合は、「０」万円としてください。

　そのうち平成23年11月にかかった平成22年５月生まれのお子さんの保育料についておたずねします。**あてはまる番号**に○をつけてください。**「保育料あり」の場合は金額を**□に記入してください。
（ここでいう「保育料」は、保育所、託児所、保育ママさんやベビーシッターなどに支払った費用。一時的に預けた費用も含む。）

1　保育料なし
2　保育料あり→ □ 万 □ 千円

※　千円未満は四捨五入してください。
　月額が500円未満は「０」千円としてください。

※　最後に、お子さんを育てておられてふだん感じていること、この調査に関することなど何でも結構ですので、自由にご記入ください。

なお、下記の記載欄に記入された内容について、個人が特定できないようにした上で、お便りや白書などでご紹介させていただくことがありますので、使用してもよろしければ□欄にチェックをお願いします。

使用可 □

SAMPLE

ご協力ありがとうございました。

最後にもう一度、記入されていないページがないかご確認のうえ、お早めに、同封の返送用封筒にてご投函ください。

なお、この調査の結果は、まとまり次第皆様のもとにお届けします。

今後ともご協力をいただきますようお願いします。

※　この調査についての連絡・問い合わせ先

厚生労働省 大臣官房 統計情報部
社会統計課 縦断調査室 出生児調査第二係

代表電話　(03) 5253-1111 (内線7566)
直通電話　(03) 3595-2321
Eメール　b-cohort@mhlw.go.jp

電話による問い合わせは、
平日の月曜～金曜の午前９時30分から
午後６時15分までにお願いします。
